# Supplementary material for: The role of emergency departments in opioid related harms: A qualitative study among emergency healthcare providers
Source: PLoS One. 2025 Dec 19;20(12):e0338421. doi: 10.1371/journal.pone.0338421 (PMC12716695; doi:10.1371/journal.pone.0338421)
Supplement: S2 File — (PDF) [file pone.0338421.s002.pdf]

TOPIC GUIDE

Qualitative study among physicians and physician assistants working in the Emergency Department regarding opioid use, misuse, and prescribing practices

This qualitative interview study aims to explore the perceptions of physicians and physician assistants (PAs) working in emergency departments (EDs) regarding opioid use, misuse, and prescribing behaviour. In addition, we aim to understand how ED professionals perceive the role of the emergency department in addressing opioid-related problems.

The interviews will last approximately 30–45 minutes. Each interview will be audio-recorded and transcribed verbatim. Data will be coded and analysed thematically. Only the direct researchers will have access to the coded data.

Participants are encouraged to elaborate on questions or topics and to share specific examples or experiences where relevant.

1. Preliminary questions

a. Do you agree that this interview will be audio-recorded, transcribed verbatim, and stored in a secure database at the LUMC? These data will be retained for 15 years.
b. Do you agree that a limited amount of personal information will be added to this database?

2. Background information

a. What is your age?
b. What is your gender?
c. What is your current position?
  i. How many years of experience do you have in this position?
d. How many years of total experience do you have in emergency medicine?
  i. At which ED are you currently employed?
  ii. How long have you worked at this ED?
  iii. At which other EDs have you worked (domestic or abroad)? What was their size?
  iv. Have you worked in other specialties? If so, which, where, and for how long?

3. Experiences with opioid prescribing

a. For which types of complaints do you typically prescribe opioids? When would you prescribe them and when not?
b. What are your thoughts or feelings when prescribing an opioid (positive or negative)? How does this differ from prescribing, for example, diclofenac?
  i. How do you think this influences your prescribing behaviour?
  ii. Does this differ when administering morphine in the ED?
c. Are there factors that make you reluctant to prescribe opioids?
  > (Probe: when and why? Patient factors such as addiction, medical history, type of complaint? Personal factors? Other considerations?)
d. Do you approach patients already using opioids differently when prescribing additional or new opioids?
  i. In terms of pain management?

ii. In terms of addiction risk?
e. Do you think opioid prescriptions are sometimes given too easily in the ED? Do you discuss this with colleagues?
f. To what extent do you think opioid prescribing in the ED can contribute to the development or maintenance of opioid-related problems, such as misuse or dependence?
g. How do you think patients perceive receiving an opioid prescription? What are your experiences?
i. Do you think some patients expect to receive opioids? How can you tell, and how does that affect you?
ii. Do you encounter patients who refuse or avoid opioids? How can you tell, and how does that affect you?

4. Experiences with patients with opioid-related problems

a. How often do you encounter patients with opioid-related problems, such as side effects, overdose, or dependence?
> (Probe: patients presenting to the ED with problems due to opioids.)
b. How do you view patients who visit multiple EDs seeking opioid prescriptions?
i. What actions do you take in such cases?
c. Do you ever consult general practitioners or psychiatric consultants when suspecting an opioid use disorder? Why or why not?
d. Do you have experience referring patients to addiction services? How was this experience?
e. Are you willing to refer patients to addiction care? Why or why not?
i. Do you think a specific protocol or contact point could help with this?
f. To what extent would you be willing to initiate treatment for opioid misuse from the ED?
i. Would you consider prescribing methadone, buprenorphine, or naloxone? Why or why not?

5. Care for patients with opioid dependence in the ED

a. What are your thoughts about this patient group?
b. To what extent do you find caring for these patients fulfilling?
c. Do you think the ED is the right setting to help these patients?
d. How do you think these patients could best be supported?
e. What do you think is currently missing in the care for this patient group?
f. (Optional) Have you had any personal experience with opioid-related problems, either directly or within your close environment?

6. The role of the ED in addressing opioid-related problems

a. To what extent do you think opioid misuse is a major public health issue at present? Have you noticed any changes in recent years?
i. What are your thoughts or feelings about this?
b. Who or what do you think is primarily responsible for opioid misuse?
> (Probe: patients, society, physicians, the pharmaceutical industry, etc.)
c. To what extent do you think the ED has a role to play in addressing opioid-related problems from a broader societal perspective?
