# Supplementary material for: The role of emergency departments in opioid related harms: A qualitative study among emergency healthcare providers
Source: PLoS One. 2025 Dec 19;20(12):e0338421. doi: 10.1371/journal.pone.0338421 (PMC12716695; doi:10.1371/journal.pone.0338421)
Supplement: Attachment — Submitted filename: The role of emergency departments in opioid related harm.docx [file pone.0338421.s004.docx]

**“The role of emergency departments in opioid related harm: A qualitative study among emergency healthcare providers”**

This study uses 25 semi-structured interviews with physicians and other providers in emergency departments in the Netherlands to understand the role of those departments in preventing and coping with opioid misuse, overdose, and addiction. The analysis and discussion are organized around two central themes that emerged from the analysis – one pertaining to preventing opioid-related harm, and the other pertaining to managing problematic opioid use, both within emergency departments. This is an important topic, particularly in the European context, where problematic opioid use and its harms are on the rise, and where research about how healthcare institutions can help prevent these outcomes among patients is timely. The article is well-written and the methodology is appropriate.

I am happy to recommend publication of this article, although I would recommend that the authors could make some small – but important – improvements before the manuscript is accepted. These recommendations are described below.

1. The Introduction rightly makes the point that the opioid misuse situation is not yet as dire in Europe as in the United States, and that these contrasts suggest the need for Europe-centered research. This particular study is based in the Netherlands – with it’s specific demographics and healthcare institutions. I would like to see the authors comment specifically on how the Dutch context – and therefore what we can learn from this study – fits within the broader European context, and the extent to which other European nations may also gain (or not) from their findings. This content should be presented in both the Introduction and the Discussion.
2. The study sites were all academic medical centers or teaching hospitals. How does this limit (or otherwise shape) the results and their interpretation and transferability? What – if anything – might be different in community-based settings that are not teaching hospitals or academic centers?
3. The authors note that data saturation was reached after 20 interviews. I would like the authors to say more about how this was determined.
4. I would like to see a few more details in the “Data Collection” section. In particular, how open-ended was the questioning? Were follow-up questions asked, and what types of questions were those? It would help to provide the interview guide – either summarize the questions in the text itself or provide the entire guide as a Table or Supplement.
5. In the “Data Analysis”, the authors cite Braun and Clarke, but I would like to hear more about specifically how the initial coding phase was completed in this study. Another sentence or two should be enough; the description should be sufficient to allow a reader to envision working directly with the data themselves.
6. The “Ethical Considerations” section currently includes recruitment information; this might be better off earlier in the Methods section. But either way, the authors should add information about how many of the healthcare providers they approached agreed to participate, and the reasons why others did not.
7. Table 2 has a complex organizational structure that is difficult to follow visually. Perhaps this could be simplified? Regardless, it would be helpful to describe the purpose of the left-most column a bit more. The Table should also be described in the text.
8. Result 1.1.2 had me wondering whether the opioid prescribing guidelines in the Netherlands currently specify anything re: prescription length, or if that is among the specifics that are not fully present in the current clinical guidance? Also on the subject of clinical guidance, it would be helpful (in terms of the policy implications) to suggest what specific entities could provide the more-thorough guidance the authors’ findings point toward.
9. Each of the Results subsections includes 1-2 participant quotes, which do a nice job of helping to illustrate the data from which the findings are generated. The quotes are always presented at the end of the subsection, though, even when they are illustrating only one of the analytic points described in the text above. I think the text might flow more easily for readers if the quotes were instead presented right after the text to which they are relevant; additional text can appear after the quote.
10. The contemporary perception that pain should not be accepted and must be treated is emphasized several times throughout the article, but section 1.3.2 also notes the converse cultural tendency (to undertreat pain). I would like to see the authors clarify throughout the manuscript whether these are balanced cultural tendencies – according to the study participants – or whether the first truly dominates.
11. Section 2.1.3 talks about the role of GPs and participant concerns about whether GPs act on the communications they send after an ED contact; I found myself wondering whether the Dutch system includes any structured way of ensuring that GPs in fact get these communications at all?
12. The authors mention the Prescription Drug Monitoring Programs (PDMPs) of U.S. states in the Discussion. As a U.S.-based scholar, I found myself thinking of the relevance of these programs much earlier, and would encourage the authors to work this into the Introduction as well. Also, some U.S. states require prescribers to consult the state PDMP before prescribing any opioids – this is relevant around line 702 of this manuscript and could be mentioned around this point.
13. The authors mention on line 719 that the lack of after-hours availability of GPs and addiction care services is expected to worsen over time in the Netherlands. Why is this?
14. Three PAs were included among the participants, most of whom were physicians. Were their perspectives distinctive in any way? I would like to see the authors comment on this regardless of the answer.
