# Supplementary material for: The role of emergency departments in opioid related harms: A qualitative study among emergency healthcare providers
Source: PLoS One. 2025 Dec 19;20(12):e0338421. doi: 10.1371/journal.pone.0338421 (PMC12716695; doi:10.1371/journal.pone.0338421)
Supplement: Attachment — Submitted filename: Rebuttal letter.docx [file pone.0338421.s005.docx]

Dear Dr. Amankwaa,

Please find enclosed our rebuttal related to our submitted manuscript with Submission ID PONE-D-25-24623, entitled *“*The role of Emergency Departments in opioid related harm: a qualitative study among emergency healthcare providers".

We would like to thank the editor and reviewers for evaluating our manuscript and for the constructive comments by the reviewers. In this rebuttal letter we address the specific comments mentioned by the editor and reviewers, we will ensure that our manuscript is fully aligned with PLOS ONE’s style and policy requirements.

We hereby submit a revised version of our manuscript, in line with the reviewers’ suggestions.

We thank you for considering our manuscript for publication in *PLOS One.*

Kind regards,

On behalf of the other authors of this manuscript,

Charlotte ten Pas

**Editor comments:**

1.Please ensure that your manuscript meets PLOS ONE's style requirements, including those for file naming. The PLOS ONE style templates can be found at

<https://journals.plos.org/plosone/s/file?id=wjVg/PLOSOne_formatting_sample_main_body.pdf> and

[https://journals.plos.org/plosone/s/file?id=ba62/PLOSOne_formatting_sample_title_authors_affiliations.pdf](https://track.editorialmanager.com/CL0/https:%2F%2Fjournals.plos.org%2Fplosone%2Fs%2Ffile%3Fid=ba62%2FPLOSOne_formatting_sample_title_authors_affiliations.pdf/1/010f0199366554a8-6a7272bd-50a7-4d3d-9e88-6b9bdb04ae75-000000/yh4IZQQlRBf_WHlBDs0facd7VP5kKKnLMxJpS-SAo6A=228)

Response: We reformatted the manuscript according to the PLOS ONE templates and ensured correct file naming conventions.

2. PLOS requires an ORCID iD for the corresponding author in Editorial Manager on papers submitted after December 6th, 2016. Please ensure that you have an ORCID iD and that it is validated in Editorial Manager. To do this, go to ‘Update my Information’ (in the upper left-hand corner of the main menu), and click on the Fetch/Validate link next to the ORCID field. This will take you to the ORCID site and allow you to create a new iD or authenticate a pre-existing iD in Editorial Manager.

Response: The corresponding author’s ORCID iD has been validated in Editorial Manager.

3. Please provide a complete Data Availability Statement in the submission form, ensuring you include all necessary access information or a reason for why you are unable to make your data freely accessible. If your research concerns only data provided within your submission, please write "All data are in the manuscript and/or supporting information files" as your Data Availability Statement.

*Response*: The qualitative interview data analyzed in this study are not publicly available due to confidentiality considerations. De-identified quotes supporting the findings are included within the manuscript.

4. We note you have included a table to which you do not refer in the text of your manuscript. Please ensure that you refer to Table 2 in your text; if accepted, production will need this reference to link the reader to the Table.

Response: We added a reference to Table 2 in the Results section (page. 11, line 326 and page. 18 line 527)

5. If the reviewer comments include a recommendation to cite specific previously published works, please review and evaluate these publications to determine whether they are relevant and should be cited. There is no requirement to cite these works unless the editor has indicated otherwise.

*Response*: We reviewed all suggested references.

**Additional Editor Comments:**

The most urgent revisions concern clarity and rigor in the **Methods** (recruitment details, interviewer role, analytic procedures, and justification of data saturation) and restructuring of the **Results** (particularly simplifying Table 2, integrating participant quotes into the text, and synthesizing themes). The **Background and Discussion** also need strengthening: situating the Dutch findings within a broader European and U.S. context, addressing the role of guidelines, stigma, and cultural attitudes to pain, and clarifying the ethical and policy implications. In addition, consistent terminology and careful attention to phrasing are expected throughout. Please ensure your manuscript is aligned with the **COREQ checklist** (or another relevant EQUATOR Network checklist such as SRQR).

Please revise your manuscript accordingly and submit a **point-by-point response document** indicating how each reviewer’s comment has been addressed. Where you disagree with a suggestion, provide a clear rationale.

*Response*: In line with the summary of the main comments of the reviewers by the editor, we revised the manuscript extensively. We respond point-by-point to each comment of the reviewers below in further detail. Overall, we addressed the comments mentioned above as follows:

We expanded the **Methods** section to include:

Recruitment details (Study Design and Setting):

We added more detail on the recruitment process, adding the following information:

‘Purposive sampling was utilized to select a diverse group of healthcare professionals, considering factors such as age, sex, profession, and years of experience, while also achieving variation across hospital types and regions.’ (Page 7 lines 227-229)

‘An invitation to participate in the study was emailed to all emergency physicians and PAs working in the four participating EDs (around 20 clinicians per site) several weeks prior to the interviews. In addition, the interviewer visited each ED on multiple occasions to facilitate recruitment. No financial or other incentives were offered. Nearly all clinicians who were approached agreed to participate; the few who did not participate cited scheduling conflicts or workload as the main reasons. Participants understood that their statements might be quoted in the manuscript.’ (Page 7, lines 230- 237)

Interviewer Role:

We added a more detailed description of the interviewer: ‘All interviews were conducted by C.t.P., a resident physician with two years of working experience in the ED. The interviewer was known within the LUMC but had no prior relationship with participants from the other three hospitals. Participants were informed that the interviewer was a resident at the LUMC ED and that the study aimed to explore physicians’ perspectives on opioid prescribing and opioid-related issues. … The interviewer's background in emergency care enriched the credibility and quality of the insights gathered. As participants interacted with someone who understood the complexity and nuances of their work environment, they were more inclined to engage in detailed discussions about their experiences and challenges.’ (Page 8, lines 241-251)

Analytic procedures (Data analysis):

We elaborated in more detail on the analytic approach, describing the coding process, the theme development, and the two thematization meetings to ensure validity and reflexivity throughout the ‘Data analysis’ section. (Page 9, lines 273-294)

Justification of data saturation (Data Collection):

We further clarified this section: ‘Data saturation was achieved after 20 interviews, as no additional significant information was captured beyond this point. To verify saturation, five additional interviews were conducted using purposive sampling to ensure diversity and increase the likelihood that new information would be uncovered. These interviews did not provide relevant new insights.’ (Page 8 lines 259-262)

*Response*: We expanded the **Results** section to include:

- Table 2: We revised Table 2 to improve clarity and reduce redundancy.
- We integrated representative participant quotes directly into the Results narrative.
- To reduce fragmentation and enhance clarity, we merged several subthemes. The subtheme 'Alternative pain medication' was combined with 'Opioid prescriptions at discharge'. Additionally, healthcare providers were grouped into a subtheme. Finally, ‘Patient knowledge about opioids’ and ‘Expectations’ were integrated into one subtheme titled 'Knowledge and expectations regarding opioids'.

*Response*: We expanded the **Background & Discussion** section to include:

- Strengthen sections by situating findings in a broader European and U.S. context (Pages 3,4 lines 102-123), (page 5,6 lines 175-199), (page 30 lines 916-920)
- Addressing the role of guidelines, stigma and cultural attitudes to pain. (page 4,5 lines 132-158), (page 27 lines 825-829), (page 28, lines 876-882)
- Clarifying ethical/policy implications. (Pages 5 lines 159-163), (page 26 lines 795-804), (page 27 lines 836-845), (page 29, lines 895-896)

**Terminology & phrasing**

Ensure consistent terminology and careful phrasing throughout.

*Response*: We revised the entire manuscript for consistency and clarity.

**Checklist compliance**

Align manuscript with COREQ (or other relevant EQUATOR checklist).

*Response*: We ensured compliance with the COREQ checklist and uploaded the completed checklist with the revised submission.

**Reviewer Comments:**

Reviewer 1

**“The role of emergency departments in opioid related harm: A qualitative study among emergency healthcare providers”**

This study uses 25 semi-structured interviews with physicians and other providers in emergency departments in the Netherlands to understand the role of those departments in preventing and coping with opioid misuse, overdose, and addiction. The analysis and discussion are organized around two central themes that emerged from the analysis – one pertaining to preventing opioid-related harm, and the other pertaining to managing problematic opioid use, both within emergency departments. This is an important topic, particularly in the European context, where problematic opioid use and its harms are on the rise, and where research about how healthcare institutions can help prevent these outcomes among patients is timely. The article is well-written and the methodology is appropriate.

I am happy to recommend publication of this article, although I would recommend that the authors could make some small – but important – improvements before the manuscript is accepted. These recommendations are described below.

1. The Introduction rightly makes the point that the opioid misuse situation is not yet as dire in Europe as in the United States, and that these contrasts suggest the need for Europe-centered research. This particular study is based in the Netherlands – with it’s specific demographics and healthcare institutions. I would like to see the authors comment specifically on how the Dutch context – and therefore what we can learn from this study – fits within the broader European context, and the extent to which other European nations may also gain (or not) from their findings. This content should be presented in both the Introduction and the Discussion.

*Response:*

We thank the reviewer for this suggestion. We have extensively adjusted the Introduction and Discussion section to reflect on the Dutch situation, in the broader European and US context.

We added to Introduction: ‘The Netherlands, with its universal health insurance and regulatory oversight, has so far succeeded in keeping opioid-related harms relatively contained. Although many European countries share similar regulatory safeguards, such as bans on direct-to-consumer pharmaceutical advertising, they often lack the Netherlands’ highly integrated primary care infrastructure. Lessons for other European healthcare systems include prioritizing robust primary care gatekeeping, implementing regular medication reviews, developing practical prescribing tools, and fostering interdisciplinary collaboration [26, 27]. However, rising trends in opioid prescriptions, hospital admissions, and mortality within the Netherlands highlight the need for ongoing vigilance. Strengthening the role of primary care in overseeing safe pain management remains essential, both nationally and across European systems seeking to address emerging opioid concerns. At the same time, differences in healthcare organization, prescribing culture, and national guidelines may limit direct generalizability to other European countries.’ (page 5 lines 175-193)

We added to Discussion: ‘We found evident variations in opioid prescribing practices across EDs between participants, particularly concerning the selection of long-acting versus short-acting formulations and prescription duration.

Cultural attitudes toward pain and opioid use differ markedly between the Netherlands and the US. In the US, aggressive pain management and pharmaceutical marketing contributed to high opioid consumption and misuse [35, 36]. In contrast, European countries, including the Netherlands, have more restrictive prescribing cultures and greater emphasis on non-opioid pain management [37].’ (page 27 lines 821-829)

We also added: ‘Tailoring opioid stewardship interventions to the organization of care, such as ensuring GPs have the time, skills, and resources for patient-centered deprescribing, can help address rising trends in opioid prescriptions, hospital admissions, and mortality.’ (page 30 lines 934-936)

2. The study sites were all academic medical centers or teaching hospitals. How does this limit (or otherwise shape) the results and their interpretation and transferability? What – if anything – might be different in community-based settings that are not teaching hospitals or academic centers?

*Response*: We clarified this in the ‘Methods-Study Design and Setting’ section:

‘The study was conducted with a purposive sample of PAs, residents, and emergency physicians working in various (academic and non-academic) EDs, located in both urbanized and less urban regions in the Netherlands.’

‘Small rural hospitals were not included in our sample. However, this type of hospitals is scarce within the Dutch healthcare system. The sample thus offers a strong reflection of current practice and captures the diversity most relevant to the national context.’ (Page 7 lines 213-223).

3. The authors note that data saturation was reached after 20 interviews. I would like the authors to say more about how this was determined.

*Response*: We clarified this in the ‘Methods-Data Collection’ section:

‘Data saturation was achieved after 20 interviews, as no new information was captured beyond this point. To ensure saturation, five additional interviews were conducted using purposive sampling, also ensuring diversity. These interviews did not provide relevant new insights.’ (Page 8 lines 257-262)

4. I would like to see a few more details in the “Data Collection” section. In particular, how open-ended was the questioning? Were follow-up questions asked, and what types of questions were those? It would help to provide the interview guide – either summarize the questions in the text itself or provide the entire guide as a Table or Supplement.

*Response*: We thank the reviewer for this suggestion. We have expanded the ‘Data Collection’ section as follows: ‘The topic guide was designed to elicit detailed responses regarding participants' views on opioid prescribing, their experiences and attitudes regarding opioid-related harms in the ED, allowing participants to elaborate freely on their perspectives. Follow-up and probing questions were asked to clarify or deepen responses when relevant.’ (Page 8 lines 255-258)

To increase transparency, we have added the full topic guide as a Supplement.

5. In the “Data Analysis”, the authors cite Braun and Clarke, but I would like to hear more about specifically how the initial coding phase was completed in this study. Another sentence or two should be enough; the description should be sufficient to allow a reader to envision working directly with the data themselves.

*Response:* We added more details throughout the ‘Methods-Data Analysis section:

‘The interviews were audio-recorded, transcribed verbatim (using Amberscript transcription software). To develop a comprehensive understanding of each physician's experience, the transcripts were read and reread thoroughly. Two researchers C.t.P. (female emergency medicine resident with no prior experience in qualitative research) and N.K., PhD, emergency physician, experienced in qualitative research) independently coded the transcripts with ATLAS.ti software (Version 23.2.3.27778) using the reflexive thematic analysis approach as described by Braun & Clarke [28]. This involved identifying inductive descriptive codes by highlighting recurring phrases within the physicians' narratives. Following the initial coding, C.t.P. and N.K. compared and discussed the codes, thereby enhancing the depth of the analysis. The codes were organized into themes over the course of two thematization meetings. In these sessions, the codes were collaboratively grouped to construct themes that reflected the content and nuances of the interview data.’ (Page 9 lines 273-284).

6. The “Ethical Considerations” section currently includes recruitment information; this might be better off earlier in the Methods section. But either way, the authors should add information about how many of the healthcare providers they approached agreed to participate, and the reasons why others did not.

*Response*: We have moved the recruitment details to earlier in the ‘Methods-Participants’ section, and added the following clarification on recruitment: ‘An invitation to participate in the study was emailed to all emergency physicians and PAs working in the four participating EDs (around 20 clinicians per site) several weeks prior to the interviews. In addition, the interviewer visited each ED on multiple occasions to facilitate recruitment. No financial or other incentives were offered. Nearly all clinicians who were approached agreed to participate; the few who did not participate cited scheduling conflicts or workload as the main reasons.’ (Page 7, lines 230-236)

7. Table 2 has a complex organizational structure that is difficult to follow visually. Perhaps this could be simplified? Regardless, it would be helpful to describe the purpose of the left-most column a bit more. The Table should also be described in the text.

*Response:* Table 2 was extensively adjusted, to improve clarity. The left column now lists the **main themes**, followed by **subthemes** that remain numbered as in the main text to facilitate cross-referencing. Further subdivisions that were previously numbered have now been integrated into the **Description** column instead, to enhance readability and avoid unnecessary complexity. This revised structure provides a clearer overview of the thematic hierarchy and a more concise presentation of the qualitative findings. According to Blignault I, Ritchie J. Revealing the wood and the trees: reporting qualitative research. Health Promot J Austr. 2009 Aug;20(2):140-5.

8. Result 1.1.2 had me wondering whether the opioid prescribing guidelines in the Netherlands currently specify anything re: prescription length, or if that is among the specifics that are not fully present in the current clinical guidance? Also on the subject of clinical guidance, it would be helpful (in terms of the policy implications) to suggest what specific entities could provide the more-thorough guidance the authors’ findings point toward.

*Response*: Information regarding the use of guidelines is provided in the Introduction, also see response to comment 3 of reviewer 1. We added ‘In the Netherlands, the Dutch Society of Emergency Physicians (DSEP) does not provide specific guidelines regarding pain management at the time of ED discharge. However, the Netherlands Society for Anesthesiology (NSA) offers a 'Generic Guideline Module for Appropriate Opioid Use,' which targets secondary healthcare settings, including EDs [19]. This guideline advocates for a maximum prescription duration of seven days and favors short-acting opioids. A recent study found that out of 33 EDs in the Netherlands, 58% follow locally developed opioid prescribing guidelines, which generally recommend short-term use of 3 to 7 days. Only 11% adhere to the national Dutch guideline, while the rest do not use any formal guideline for prescribing opioids at discharge. Around 40% of these local guidelines lack specific patient education, and when provided, mainly cover side effects and rarely mention addiction risks [20]. In contrast, following the severe opioid crisis, in the US more comprehensive guidelines have been developed for opioid prescribing in EDs. Recent guidelines from the Centers for Disease Control (CDC) and the American College of Emergency Physicians (ACEP) suggest a 'short duration' of opioid therapy, emphasizing clinical judgement and shared decision-making over strict limits on prescription duration [21, 22]. (Page 4-5 line 139-158)

We also added to the original 1.1.2 part, now called ‘Opioid prescriptions at ED discharge’: ‘Currently, just over half of Dutch EDs follow prescribing guidelines for pain management after discharge. Most rely on locally developed protocols, but these guidelines usually lack condition-specific recommendations [20].’ (Page 14 line 375-377)

9. Each of the Results subsections includes 1-2 participant quotes, which do a nice job of helping to illustrate the data from which the findings are generated. The quotes are always presented at the end of the subsection, though, even when they are illustrating only one of the analytic points described in the text above. I think the text might flow more easily for readers if the quotes were instead presented right after the text to which they are relevant; additional text can appear after the quote.

*Response:* We appreciate this constructive suggestion. We have revised the Results section to integrate participant quotes directly after the points they illustrate, rather than grouping them at the end of each subsection. We also added illustrative quotes in table 2.

10. The contemporary perception that pain should not be accepted and must be treated is emphasized several times throughout the article, but section 1.3.2 also notes the converse cultural tendency (to undertreat pain). I would like to see the authors clarify throughout the manuscript whether these are balanced cultural tendencies – according to the study participants – or whether the first truly dominates.

### *Response;* We added to the Results section, originally 1.3.2 Pain in society, now only pain in society:

*‘*Participants described ambivalence concerning two contrasting cultural perspectives on pain management. Some emphasized a traditional, more stoic approach to pain, characterized by the belief that pain is something to be endured rather than eliminated. This may contribute to undertreatment. Others highlighted the idea that pain should not be accepted and must be actively treated, supported by initiatives such as including pain as a (fifth) vital sign in triage protocols. This may contribute to overtreatment. These contrasting tendencies coexist within Dutch emergency medicine, reflecting broader societal and professional debates about what constitutes appropriate pain management.’ (Page 17 lines 499-511)

Further, the following text was added to the Introduction: ‘Historically, in the Netherlands, opioid prescriptions are generally characterized by lower dosages and a preference for non-opioid analgesics within multimodal pain management [9, 12]. Conversely, in the US opioids have been prescribed at higher doses and for longer durations, with a preference for potent opioids such as oxycodone as first-line therapy, often prioritizing patient satisfaction over prescribing restraint [13]. (Page 4 lines 119-123)

11. Section 2.1.3 talks about the role of GPs and participant concerns about whether GPs act on the communications they send after an ED contact; I found myself wondering whether the Dutch system includes any structured way of ensuring that GPs in fact get these communications at all?

*Response:* Thank you for this comment. We added in the (originally 2.1.3) section ‘**Perceived roles of other healthcare providers,** *The role of general practitioners*’: ‘In the Dutch healthcare system, general practitioners (GPs) routinely receive electronic discharge letters from the emergency department. However, several participants noted that while these communications are reliably transmitted, the large volume of correspondence makes it difficult for GPs to read and process every letter in detail. This contributes to concerns about whether follow-up recommendations are consistently acted upon.’ (Page 21 lines 638-643)

12. The authors mention the Prescription Drug Monitoring Programs (PDMPs) of U.S. states in the Discussion. As a U.S.-based scholar, I found myself thinking of the relevance of these programs much earlier, and would encourage the authors to work this into the Introduction as well. Also, some U.S. states require prescribers to consult the state PDMP before prescribing any opioids – this is relevant around line 702 of this manuscript and could be mentioned around this point.

*Response:* Thank you for this valuable suggestion. We now mention U.S. Prescription Drug Monitoring Programs (PDMPs) in the Introduction: ‘In response to rising opioid misuse, several countries have implemented monitoring systems to improve prescribing oversight. **In many US states, prescribers are required by law to consult the prescription drug monitoring program (PDMP) before issuing an opioid prescription.** Comparable national initiatives are largely absent in Europe, where efforts to reduce opioid-related harms have focused more on clinical guidance and professional awareness rather than prescription surveillance [9].’ (Page 4 lines 132-138)

13. The authors mention on line 719 that the lack of after-hours availability of GPs and addiction care services is expected to worsen over time in the Netherlands. Why is this?

*Response:* Thank you for this helpful question. We have clarified this in the Discussion: ‘.. It is expected to worsen over time as the national shortage of GPs increases, driven by population aging, higher care demands, and limited workforce capacity. Addiction care services are generally unavailable outside regular working hours, leaving the ED as the only accessible point of contact for many patients, restricting timely access to specialized support for patients with opioid-related problems.’ (Page 30 lines 916-920)

14. Three PAs were included among the participants, most of whom were physicians. Were their perspectives distinctive in any way? I would like to see the authors comment on this regardless of the answer.

*Response:* We would like to clarify that while three physician assistants (PAs) were included among the participants, their perspectives were consistent with those of the physicians. Because this is a qualitative study, no statistical comparisons were made between groups. PAs in the participating hospitals manage the same patient population as emergency physicians, and the additional interviews included one with a PA at the end of data collection, which did not yield new insights, confirming data saturation. (see Methods-Data Collection, page 8 lines 259-262)

**Reviewer 2:**

Thank you for the opportunity to review this manuscript, The role of Emergency Departments in opioid related harm: a qualitative study among emergency healthcare providers. This is the first qualitative study with ED providers in Europe and an important contribution to understanding the attitudes of providers in management opioid-related harms during an epidemic. I have made recommendations for the authors to strengthen this manuscript, including providing a more detailed Background with Netherlands-specific context that sets the stage for their recommendations, revising the format of Table 2, synthesizing their findings and shortening the presentation, and explaining how their findings are similar to/different from a comparable US-study.

## Abstract

1. Line 50: I would substitute “with” for “among”

*Response*: This was adjusted accordingly.

## Body

1. What do you mean by, “partly due to differences in healthcare systems”? Are you saying that opioid-related harms have been constrained in the Netherlands due to differences in the HC system, **compared t**o the US healthcare system? There seems to be an implicit comparison of these two nations. Which differences? Can you quantify them? What are examples of stricter prescribing and advertising regulations? This seems to set up your manuscript as a tension, but there is not enough information for the reader.

*Response:* We added an extensive reflection of the differences in healthcare systems to the Introduction: ‘The Netherlands' universal healthcare coverage, high population density, and swift access to emergency services may help prevent the development of opioid misuse and overdose by facilitating early intervention and comprehensive care. The healthcare system is designed to ensure equal access for all citizens, offering comprehensive insurance in a regulated market that includes free primary care. Every Dutch citizen is registered with a general physician (GP), who is typically the first point of contact for health problems. Additionally, after-hours care is managed through large-scale, uniform GP cooperatives that cover 99% of the population. These cooperatives are available from 5 p.m. to 8 a.m. on weekdays and around the clock on weekends, using a nationally uniform model with telephone triage and physicians always present on-site [7]. In contrast, the US healthcare system is characterized by greater fragmentation, variable access to care, and less centralized oversight[8].

The Netherlands and most European countries have strict regulations prohibiting direct-to-consumer advertising of prescription medications and tight controls on pharmaceutical marketing to prescribers. The Netherlands also has centralized and regulated dispensing, with most opioids prescribed in general practice and under stricter monitoring [9]. In contrast, the US healthcare system has allowed marketing and direct-to-consumer pharmaceutical advertising by pharmaceutical companies, which has been thought to have contributed to higher opioid prescribing and opioid-related harms [10,11]. Historically, in the Netherlands, opioid prescriptions are generally characterized by lower dosages and a preference for non-opioid analgesics within multimodal pain management [9,12]. Conversely, in the US opioids have been prescribed at higher doses and for longer durations, with a preference for potent opioids such as oxycodone as first-line therapy [13].’ (Page 3,4 lines 102-123)

2. Similarly, Lines 123-124: You tell us that these are different in the Netherlands, but you haven’t told us different from whom or provided examples of these differences to illustrate.

*Response:* We added a more detailed explanation about the differences in the health care systems between The Netherlands and Europe/the US to the Introduction, as described above. (Page 3,4 lines 102-123, pag 4 lines 132-142, pag 5 lines 153-158, pag 6 lines 175-199)

3. Does the Netherlands have opioid prescribing guidelines for ER physicians, and what direction do they provide? Some context would be helpful for your ms.

*Response*: This is clarified in the Introduction: ‘In the Netherlands, the Dutch Society of Emergency Physicians (DSEP) does not provide specific guidelines regarding pain management at the time of ED discharge. However, the Netherlands Society for Anesthesiology (NSA) offers a 'Generic Guideline Module for Appropriate Opioid Use,' which targets secondary healthcare settings, including EDs [19]. This guideline advocates for a maximum prescription duration of seven days and favors short-acting opioids. A recent study found that out of 33 EDs in the Netherlands, 58% follow locally developed opioid prescribing guidelines, which generally recommend short-term use of 3 to 7 days. Only 11% adhere to the national Dutch guideline, while the rest do not use any formal guideline for prescribing opioids at discharge. Around 40% of these local guidelines lack specific patient education, and when provided, mainly cover side effects and rarely mention addiction risks [20]. In contrast, following the severe opioid crisis, in the US more comprehensive guidelines have been developed for opioid prescribing in EDs. Recent guidelines from the Centers for Disease Control (CDC) and the American College of Emergency Physicians (ACEP) suggest a 'short duration' of opioid therapy, emphasizing clinical judgement and shared decision-making over strict limits on prescription duration [21, 22].‘ (Page 4,5 lines 139-158)

4. Lines 87, 94, 98: You used both “opioid-related harms” and “harm”. Please use the plural form consistently in your manuscript, with “are” not “is.”

*Response*: This was adjusted accordingly all over the manuscript.

5. Line 87: I would suggest revising to: “opioid-related harms, such as overuse and dependency, addiction, and overdose (fatal and non-fatal).” I think you could also say more here about the downstream problem of diversion to street opioids after prescriptions end, and related risks of using an unsafe (adulterated) supply, as well as injection drug use risks (HIV, hep B/C, abscesses.)

*Response*: This was adjusted accordingly, in the Introduction. We added ‘[… including opioid-related harms, such as overuse and dependency, addiction, and overdose (fatal and non-fatal). Beyond these direct harms, the discontinuation of prescription opioids can lead to diversion toward illicit street opioids, increasing exposure to unsafe or adulterated drug supplies and associated injection drug use risks (HIV, hepatitis B/C, abscesses.)’ (Page 3 lines 86-89)

6. What proportion of patients in the Netherlands who are prescribed opioids in the ED setting then develop dependency and divert to street supply? I think some quantification of “how big” this problem is, if it’s been measured, would be helpful.

*Response:* There is no published data in the Netherlands that quantifies the proportion of patients who are prescribed opioids in the ED and subsequently develop a dependency or divert those medications to illicit/’street’ supply. We are currently investigating this knowledge gap.

7. Line 97: “Since” means “Since the event/time of.” A better choice here is “Because.”

*Response*: This was adjusted accordingly.

8. Line 106: “Further, since opioid-related harm is frequently encountered in Eds.” This is confusing. What do you mean? The discussion to this point has been about post-ED prescribing opioid-related harms. What are the harms encountered in EDs? How are these harms different? Are these patients who have experienced opioid-related overdose? I see this comes a bit later (Lines 113-117), but that discussion needs to move up here, so we understand what you mean. We also need some numbers illustrating what proportion of patients are seen each year in the ED for overdoses or related treatment. In other words, what is the burden for ED physicians? Give us a sense.

*Response:*  This is clarified in the Introduction: ‘Further, EDs encounter a range of opioid-related harms, including opioid seeking, withdrawal, overdoses, and injection-related complications.’ (Page 3 lines 86-89) Further, the following numbers were added: ‘A recent Dutch study found that 15% of patients presenting at an ED were currently using prescription opioids, of which 23% exhibited signs of misuse and 10% fulfilled criteria for OUD [15]’. (Page 5 lines 159-163)

9. Lines 120-121. What is problematic opioid use? This is the first time I see this term. If you are using it to refer to both existing opioid use challenges **and post-ED prescribing harms,** you need to state this by introducing it earlier and defining it clearly.

Response: This was adjusted to opioid-related harms throughout the manuscript.

10. Line 122: Which guidelines? Do they exist? Do they need to be developed? Who would they be for? Same for educational initiatives. Provide context for your readers by describing the practice landscape in the Netherlands and what professional guidance and regulation currently exists.

*Response:* Thank you for your comment. See response to comment 8 of reviewer 1 and the response to comment 3 earlier about the guidelines and education.

11. Lines 147-56: We need more on recruitment. What materials or approaches did you use? Were these print? E.g. posters? Social media? Email? Listservs? How long was the recruitment period? Did participants receive any incentives? Did you have a target and did you meet it? If not, how did you decide on your sample size?

*Response:* Thank you for this comment. We have expanded the ‘Methods-Participants’ section to provide more details on recruitment: ‘An invitation to participate in the study was emailed to all emergency physicians and PAs working in the four participating EDs (around 20 clinicians per site) several weeks prior to the interviews. In addition, the interviewer visited each ED on multiple occasions to facilitate recruitment. No financial or other incentives were offered. Nearly all clinicians who were approached agreed to participate; the few who did not participate cited scheduling conflicts or workload as the main reasons.’ (Page 7 lines 230-236)

About the target and sample size: We have added a short explanation of how data saturation informed our sample size. In the revised Methods section, we now clarify that we continued recruitment and interviews until no new themes or insights emerged from the data, indicating thematic saturation. This approach is consistent with established qualitative research methodology, where the final sample size is determined by the point at which additional interviews no longer contribute new information. (page 8, lines 259-262)

12. Lines 172-173: What difference do you think it made for a resident to conduct these interview, compared to having a non-clinician conduct them? Do you think it helped or hindered, and how? Was this a deliberate choice? Is the resident a colleague of any of these study participants? A peer, or much younger? If so, how did you handle this? I think readers might want to know how were attentive to these methodological choices, as they have power ramifications and impact the data you collect. I am not suggesting a laborious explanation here some reflection to show you are aware that these are not minor considerations.

*Response:* We have added a reflection on the interviewer’s role in the new Methods section- Interviewer Role: ‘All interviews were conducted by C.t.P., a resident physician with two years of working experience in the ED. The interviewer was known within the LUMC but had no prior relationship with participants from the other three hospitals. Participants were informed that the interviewer was a resident at the LUMC ED and that the study aimed to explore physicians’ perspectives on opioid prescribing and opioid-related issues. They had the possibility to ask additional questions before the start of the interviews, and were assured that their input would be used solely to improve understanding of the topic. The interviewer's background in emergency care enriched the credibility and quality of the insights gathered. As participants interacted with someone who understood the complexity and nuances of their work environment, they were more inclined to engage in detailed discussions about their experiences and challenges.’ (Page 8 lines 240-251)

13. Lines 178-79: insert “and” and remove the comma

*Response:* we changed the sentence.

14. Lines 179-190: You need more here to illustrate rigor. Did the two researchers conducting the analysis have previous experience in qualitative data analysis? Was one a senior qualitative researcher? How did they reconcile disagreements? Did they validate their codebook by test coding a sample of the interviews and comparing results? Were other team members involved in this analytical work? For example, did team members review the codes or themes?

*Response:* We added more detail to the Methods-Data Analysis section: ‘The interviews were audio-recorded, transcribed verbatim (using Amberscript transcription software). To develop a comprehensive understanding of each physician's experience, the transcripts were read and reread thoroughly. Two researchers C.t.P. (emergency medicine resident with no prior experience in qualitative research) and N.K., PhD, emergency physician, experienced in qualitative research) independently coded the transcripts with ATLAS.ti software (Version 23.2.3.27778) using the reflexive thematic analysis approach as described by Braun & Clarke (16). This involved identifying inductive descriptive codes by highlighting recurring phrases within the physicians' narratives. Following the initial coding, C.t.P. and N.K. compared and discussed the codes, thereby enhancing the depth of the analysis. The codes were organized into themes over the course of two thematization meetings. In these sessions, the codes were collaboratively grouped to construct themes that reflected the content and nuances of the interview data. The first meeting took place after the first 15 interviews were conducted and included researchers C.t.P., N.K., A.S. (professor in addiction psychiatry, experienced with qualitative study methods), H.E. (PhD candidate with a background in qualitative research on opioid misuse) and V.B. (medical student / PhD candidate in research on opioid misuse). The topic guide was refined during this initial meeting, adding questions about personal experiences with opioid-related harms to deepen the discussions.’ (Page 9 lines 273-290)

15. Lines 200-202: Did the consent process include an explicit opt-in to quote participants?

*Response:* Yes, this was added to the Methods-Participants section: ‘ Participants understood that their statements might be quoted in the manuscript.’ (Page 7 line 236)

16. Line 219: Over a quarter of your sample worked “abroad.” Can you say more? Which countries? Europe only? Or beyond?

*Response:* This was clarified in the Results section: ‘Twelve out of twenty-five (48%) had previously worked at another ED, and seven (28%) participants had prior clinical experience outside the Netherlands, mainly within Europe, and a few in the US.’ (Page 10 lines 317)

17. Line 232, Table 2: The table is an unusual choice for presentation of findings in a qualitative study. It is unreadable. I strongly recommend you revise it. The three-level numbering is hard to understand and distracts from reading through the content. Select a model in other publications / other journals to use.

*Response:* Table 2 was extensively adjusted, to improve clarity.

18. Line 254: “was the lack of follow-up once the patient leaves the ED.” I imagine there is a lot here that your participants discussed. Can you elaborate? This points to concerns about the structure of the health care system, possible feelings of responsibility (guilt?) of providers, and so on. I think this is rich area, if your data support exploring this in more detail.

*Response*: We expanded this section with a quote to provide more context on participants’ reflections regarding the lack of follow-up after ED visits: ‘Several participants expressed discomfort about this discontinuity, noting that it left them uncertain about whether their actions had contributed to ongoing opioid use or dependency. (Page 13 lines 354-358)
Participant #10: “After the ED visit, I have no visibility on my patients anymore. I don’t get feedback on the actions I took, and I have no idea about their follow-up.”

19. Lines 257-259 and elsewhere: Quotes need to be integrated into the narrative, not placed at the bottom stand-alone. Please revise each section so that quotes are integrated into your presentation of findings, either with a colon and then indenting the quote, using “For example,…”, or sandwiching.

*Response:* We appreciate this constructive suggestion. We have revised the Results section to integrate participant quotes directly after the points they illustrate, rather than grouping them at the end of each subsection.

20. Lines 269-272: This is fascinating (and not unique to your population). Can you say more in your Discussion? What shapes differences in opinion? Training? Speciality? Age? Gender? Past experience? How do these differences become conflictual, either in patient hand offs or w/ the institution’s policies?

*Response:* We appreciate this insightful comment. However, we believe that our qualitative sample is not designed to support subgroup comparisons based on characteristics such as training, specialty, age, or gender. The primary goal of this study was to explore the range and reasoning behind physicians’ attitudes rather than to explain variation between subgroups. While differences in experience or background may indeed shape prescribing preferences, our data were not collected or powered to make such distinctions. We have therefore chosen to acknowledge this as a potential area for future research rather than speculate beyond the scope of our findings.

21. Lines 286-287: Revise so that you are not ending this sentence with a preposition. Also, why didn’t your sample always ask about a history? Is this due to lack of time, interest or curiosity, guidelines, institutional policy, training?

*Response*: We changed it into: ‘However, participants noted that this history is not always explored in practice, often due to time constraints in the emergency setting or perceived lack of relevance.’ (pagw 15 lines 400-402)

22. Lines 293-294: I think you need to say more about this in your Discussion. In the US, pain as the fifth vital sign, which gained acceptance through Dr. James Campbell in his 1995 presidential address to the American Pain Society, has been discredited as not evidence-based, although it is still practiced in taking a history. It is identified as a key contributor to the opioid epidemic(s) in the US, and there are excellent analyses published. It illustrates the immense influence of powerful individual physicians in shaping practice and ideology on pain management. So what is different in the Netherlands? Is this institutionalized and enshrined in practice? Has it been subject to criticism as it has in the US? Is it controversial?

*Response*: We added the following text to the Discussion: ‘Unlike in the US, where pain has been institutionalized as the “fifth vital sign,” this concept is not embedded in routine clinical practice in the Netherlands. In Dutch EDs, pain is incorporated into the Manchester Triage System as a clinical discriminator used to assess urgency, rather than being classified as a formal vital sign [30]. Standardized pain scores are commonly used for patients able to self-report, but nurses’ clinical judgment often guides management [30].Participants described a growing awareness of the importance of pain management, yet also noted differing views: some emphasized the need for proactive pain relief, whereas others highlighted that not all pain can or should be eliminated. This ambivalence reflects ongoing debate in Dutch emergency medicine about balancing adequate analgesia with cautious opioid prescribing.’ (Page 26 lines 796-804)

23. There are too many subthemes. It leads to a cut-up presentation of findings that is overly long. Synthesize your findings thoughtfully to draw out what is relevant, and avoid presenting them in multiple choppy sections.

*Response:* Thank you for this helpful feedback. We agree that the initial structure contained too many subthemes, which fragmented the presentation of findings. We have now streamlined the Results section to improve readability and coherence. Specifically, we removed the numbers in the subthemes, merged and reorganized several subthemes:

Subtheme **1.1.3** has been integrated into **1.1.1** to avoid redundancy, now called ‘Responsibilities in prescribing opioids’

Subthemes **1.1.5** and **1.1.6** have been combined under the new subtheme ‘Perceived role of other healthcare providers.’

Subthemes **1.2.1** and **1.2.2** have been merged into ‘Knowledge and expectations regarding opioids’, and parts of **1.2.2** have been integrated with **1.3.2** (‘pain in society’) for thematic consistency.

In Theme 2, subthemes **2.1.3–2.1.5** have been merged into a single subtheme titled ‘Perceived role of other healthcare providers.’

This restructuring reduces repetition and results in a more focused and cohesive narrative of the findings.

24. Your Discussion is quite nice and integrates the findings. Please address the questions I asked above to provide some elaboration and depth. Additionally, stigma among providers is well known and shapes prescribing attitudes. I saw it mentioned in passing once. Please elaborate on the role you think it has among your study populations and their attitudes in your Discussion.

*Response:* The following text was added to the Discussion*: ‘*Stigma surrounding substance use was recognized as a possible influential factor shaping clinicians’ attitudes and prescribing decisions. Several participants noted that a history of substance use was often not explicitly explored during ED encounters, potentially reflecting discomfort around the topic. This stigma may contribute to underrecognition of opioid misuse risk and missed opportunities for early intervention. Addressing this requires targeted education and communication training to normalize discussions about substance use and promote nonjudgmental, patient-centered prescribing practices.’ (Page 28 lines 876-882)

We also added: ‘It was also noted that when a patient’s record indicated possible opioid misuse or “shopping,” clinicians sometimes preferred to consider non-opioid alternatives first, suspecting drug-seeking behavior. While not entirely unjustified, this represents a difficult clinical and ethical balance in acute pain management. (page 29 lines 888-891)

25. While this is the first investigation of its kind in Europe, a comparable study has been published in the US, on pain management in the ED. <https://pmc.ncbi.nlm.nih.gov/articles/PMC6124837/> Please incorporate this study in the Background and Discussion. How are your finding similar? What is unique to your sample and setting?

*Response:* Our study aligns with Sinnenberg et al.'s findings in several ways. This is added to the Discussion: ‘Notably, our findings on opioid prescribing at ED discharge are largely consistent with those reported in a qualitative U.S.-based study from 2017 on the same topic, reflecting similar clinical considerations [38]. However, U.S. physicians frequently referenced patient satisfaction scores as a factor influencing prescribing decisions, a consideration not present in our study in the Dutch healthcare context, where such metrics are not routinely used. Furthermore, opioid prescribing in U.S. EDs is subject to stricter regulatory frameworks, including state-level legislation that imposes limits on amounts and restricts the types of opioids that may be prescribed. In this regard, Dutch emergency physicians operate with greater clinical autonomy [1, 22, 38]’ (Page 27 lines 836-845)

26. Lines 679-687: I think this needs to be moved to your Background, and then you can introduce the Netherlands context. Guidelines are like the ghost in the room in this manuscript: they are clearly relevant but hidden just out of sight, a kind of phantom presence. If one of your messages is that guidelines in the Netherland (and you haven’t been identified these) need to be strengthened, then be explicit about this in your Background and come back to it as a recommendation. Similarly, with PDMPs. The place to introduce these is the Background, not the Discussion. Then you can come to them in the Discussion: what do your findings suggest about these? Your narrative is confusing—I can’t tell why you’re returning to the US context when the study is in the Netherlands. You seem to suggest, rather tentatively, that the Netherlands should adopt these. Without this discussion in the Background, that (1) the Netherlands doesn’t have them, and (2) why it does not, introducing them here does not make sense. Set this up more clearly so your recommendation flows from the start. And please be confident in your recommendations.

*Response:* We appreciate this insightful comment. We have revised the Background to more clearly introduce the status of opioid prescribing guidelines and the absence of a national prescription monitoring system in the Netherlands. These contextual details now help frame our findings and strengthen the rationale for our recommendations (Page 4 lines 132-158). In the Discussion, we explicitly return to these topics, linking our findings to the need for clearer national guidance on opioid prescribing and consideration of structured monitoring tools to enhance safe use. (Page 28 lines 879-882, page 29 lines 893-896, page 30 lines 934-936)

27. Do you think that your manuscript is about ethical dilemmas in managing opioid-related harms among ED providers? The word “ethical” is not present, yet this to me is the core of what you are addressing. There is a bioethics literature that you could link to that might provide more scaffolding for your findings.

*Response*: We thank the reviewer for highlighting this point. We agree that the issues described in our study such as balancing pain relief with the potential risk of opioid misuse, can certainly have ethical dimensions. However, our participants did not explicitly frame their experiences in ethical or moral terms, and our analysis was not conducted with an ethical vision. We therefore prefer not to reframe the study as one primarily about ethical dilemmas. Instead, we have clarified in the Discussion that some of the tensions described by participants may implicitly reflect underlying moral considerations in clinical decision-making and about stigmas. We believe this approach best reflects both the data and the study’s scope, while leaving room for future research to explore these questions from an ethical perspective.
